# Exploring Balance Impairment and Determinants in Chronic Obstructive Pulmonary Disease: A Comparative Study with Healthy Subjects

**DOI:** 10.3390/diagnostics14141489

**Published:** 2024-07-11

**Authors:** Hikmet Ucgun, Meltem Kaya, Hamza Ogun, Hilal Denizoglu Kulli

**Affiliations:** 1 Department of Physiotherapy and Rehabilitation, Faculty of Health Sciences, Istanbul Atlas University, Istanbul 34408, Turkey; meltem_rmglu@hotmail.com (M.K.); hilal_denizoglu_7@hotmail.com (H.D.K.); 2 Department of Chest Diseases, Faculty of Medicine, Bezmialem Vakif University, Istanbul 34093, Turkey; hamzaogun@gmail.com

**Keywords:** balance, chronic obstructive pulmonary disease, functional capacity, inspiratory muscle strength, peripheral muscle strength

## Abstract

**Background:** Many pulmonary and extrapulmonary factors may impair balance in patients with chronic obstructive pulmonary disease (COPD), but the determinants of this impairment are still debated. The primary aim was to compare both balance-related and independent variables that may affect balance with healthy subjects. The secondary aim was to investigate the potential determinants of balance in patients with COPD. **Methods:** This comparative study recruited 23 patients with COPD and 23 age- and comorbidity-matched healthy subjects. Participants were assessed regarding demographic and clinical data, “Postural Stability Test” (PST), “Limits of Stability Test” (LOST), “Clinical Test of Sensory Integration of Balance” (CTSIB), pulmonary function, respiratory and peripheral muscle strength, functional capacity, and cognitive function. **Results:** There were significant differences in all outcome measures assessing balance, pulmonary function, respiratory muscle strength, peripheral muscle strength, and functional capacity, but not cognitive function, in the COPD group compared to the healthy group (*p* < 0.05). The PST had a significant and strong correlation with maximal inspiratory pressure (MIP) (r = −0.630, *p* = 0.001) and a significant and moderate correlation with m. quadriceps strength and 6 min walk test (6MWT) distance (r = −0.472, *p* = 0.023; r = −0.496, *p* = 0.016; respectively). MIP, m. quadriceps strength, and 6MWT distance were independent predictors to explain the PST with an R^2^ = 0.336 (*p* = 0.004). **Conclusions:** The present study revealed that balance is impaired in adults with COPD, even if compared with age- and comorbidity-matched healthy subjects. Assessing and improving balance and its determinants, inspiratory and peripheral muscle strength, and functional capacity may be important for fall prevention and disease management in patients with COPD.

## 1. Introduction

Chronic obstructive pulmonary disease (COPD) was defined in “The Global Initiative for Chronic Obstructive Lung Disease (GOLD) 2023 Report” as a heterogeneous lung disease characterized by pulmonary features (dyspnea, cough, expectoration, and exacerbations) due to abnormalities of the airways and/or alveoli that cause persistent and progressive airflow obstruction [1]. The global prevalence of COPD corresponds to approximately 12% of the world population. COPD is currently the third cause of death in the world, and it is estimated that there may be more than 5.4 million deaths annually from COPD by 2060 [2]. The systemic effects of COPD lead to various extrapulmonary features that may be related to muscle dysfunction, such as inactivity, physical deconditioning, poor cognitive function, and balance [3,4]. In a recent meta-analysis, it was stated that muscle power and related physical parameters may be impaired in patients with COPD due to various demographic and pathological factors, but the basic pathophysiological mechanism has not been clearly explained [5].

Impaired balance could constitute significant morbidity leading to a loss of functional independence and falls. A recent meta-analysis reported that the prevalence of falls in patients with COPD was 30% [6]. Although falls seem potentially less significant than the pulmonary effects of the disease, they are strongly associated with higher mortality rates, reduced independence, and worsened quality of life in patients with COPD [4]. Systemic inflammation, impairment of proprioceptive control, skeletal muscle dysfunction, and decreased exercise capacity and trunk and respiratory muscle coordination have been reported as factors that may disrupt balance in COPD [4,7]. Various clinical assessments have been suggested to assess both static and dynamic balance impairment in patients with COPD. Single leg stance, the Timed Up and Go test, and the Berg Balance Scale are the most commonly used balance assessment methods in patients with COPD, and the results of these tests have shown conflicting results regarding balance impairment compared to healthy controls and variation in impairment according to disease characteristics. The fact that these tests do not measure static and dynamic balance with specific and objective methods based on postural sway and indirectly assess the risk of falling may be effective in the variability of the results [3].

It is hypothesized that the decrease in both respiratory and peripheral muscle strength may affect balance by impairing the function of the core and extremity muscles that provide postural control, resulting in a decrease in physical activity and functional capacity [4]. Cognitive impairment, which may be caused by systemic inflammation, nocturnal desaturations, and hypercapnia, has been reported in patients with hypoxic COPD. The impact of cognitive function on balance primarily depends on the difficulty level of the cognitive task, and postural sway increases linearly with the difficulty of the cognitive task [8]. What is not yet clear is whether possible variables that may affect balance are indeed impaired compared to healthy individuals and whether these variables are related to a specific testing method that assesses balance in terms of static and dynamic balance [3,8,9,10]. Although many studies have reported possible factors that play a role in the deterioration in balance in patients with COPD, it has been stated that there is a need for studies in which pulmonary and extrapulmonary features are assessed together and related factors are investigated with a multiple linear regression model [3,4].

To the best of our knowledge, there is no study in which balance in patients with COPD is assessed with sensitive and specific laboratory-based tests and all potential pulmonary and extrapulmonary features that may impair balance are assessed together and their relationship is investigated. Thus, considering the importance of balance in patients with COPD, the primary aim of this study was to assess static and dynamic balance with an objective and reliable measurement method and to compare both balance-related and independent variables that may affect balance with age- and comorbidity-matched healthy subjects. The secondary aim of this study was to investigate the potential determinants of balance in patients with COPD.

## 2. Material and Methods

### 2.1. Study Design and Subjects

A prospective, comparative study was conducted. Twenty-three patients diagnosed with COPD who were referred from the outpatient clinic of the Department of the Chest Diseases Faculty of the Medicine Bezmialem Vakif University and twenty-three age- and comorbidity-matched healthy subjects were recruited for the study between January 2023 and May 2023. The inclusion criteria of the patients with COPD were as follows: diagnosed with COPD in accordance with the GOLD criteria [1], being the ages of 40 and 65 years, and being able to read written and understand spoken language. The patients who were unable to perform tests because of diagnosed comorbidities, had an exacerbation in the last 8 weeks, and were treated with long-term oxygen were excluded. The inclusion criteria of the healthy subjects were being the ages of 40 and 65 years and being able to read written and understand spoken language. The healthy subjects who were unable to perform tests because of diagnosed comorbidities were excluded. This study was approved by the Biruni University Ethics Board (approval number: 2023/78-27), and this study was registered to the ClinicalTrials.gov website (registration number: NCT05771506). This study was conducted based on the ethical principles for human research as outlined by the Declaration of Helsinki and written informed consent was obtained from all participants.

### 2.2. Outcome Measures

**Figure 1:**
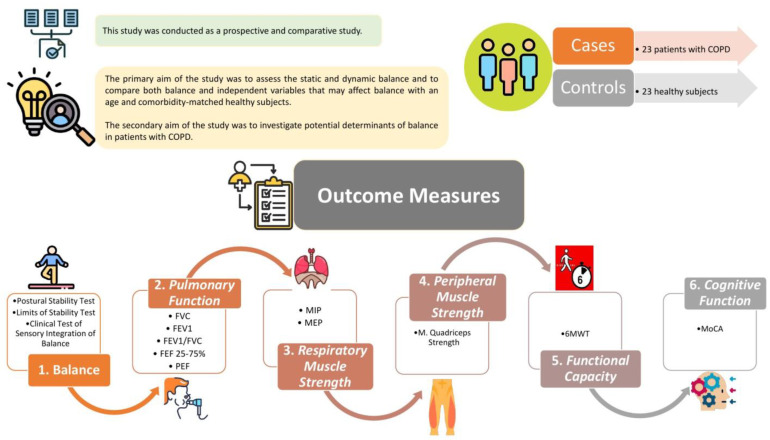
Method section of the study.

Demographic and clinical characteristics of the participants were recorded. The participants performed “Postural Stability Test” (PST); “Limits of Stability Test” (LOST); “Clinical Test of Sensory Integration of Balance” (CTSIB); spirometry, maximum inspiratory (MIP), and expiratory (MEP) pressure measurements; knee extensor strength test; 6 min walking test (6MWT); and Montreal Cognitive Assessment (MoCA) test, respectively, on the same day with adequate rest intervals (Figure 1).

#### 2.2.1. Balance

The PST, LOST, and CTSIB were assessed with the valid and reliable device Biodex Balance System^®^ (BBS; Biodex Medical Systems, Shirley, New York, NY, USA) [11]. In the PST, subjects are instructed to remain still on the platform, and the displacement of the center of gravity in the anterior, posterior, and medial–lateral axes is measured. The overall score of PST was recorded. Higher scores indicate worse static postural stability. In the LOST, subjects are instructed to move across the platform in eight directions to try to move the cursor displayed on the screen within the target circles. The overall score of LOST was recorded. Higher scores mean better dynamic postural stability. In the CTSIB, subjects are asked to maintain a fixed posture in four different positions: eyes open firm surface, eyes closed firm surface, eyes open foam surface, and eyes closed foam surface. The composite score of CTSIB was recorded. Higher scores indicate that the deterioration in balance is higher.

#### 2.2.2. Pulmonary Function

The pulmonary function test (PFT) was conducted following the guidelines of the American Thoracic Society (ATS) and the European Respiratory Society (ERS) using a spirometer (COSMED Pony FX; Rome, Italy) [12]. The forced vital capacity (FVC), forced expiratory volume in 1 s (FEV1), FEV1/FVC, peak expiratory volume measured at 25% and 75% of forced expiratory time (FEF 25–75%), and peak expiratory volume (PEF) were measured and expressed as percentages of the predicted values.

#### 2.2.3. Respiratory Muscle Strength

A MicroRPM (Micromedical; Rochester, UK) was used to measure MIP and MEP values in accordance with ATS/ERS criteria [13]. The highest value of three efforts that vary less than 5% was recorded for inspiratory and expiratory pressures.

#### 2.2.4. Peripheral Muscle Strength

The MicroFet2 hand-held dynamometer (Hoggan Health Industries, Inc.; West Jordan, UT, USA) was used with the breaking method to assess knee extensor muscle strength. The maximum value of three consecutive measurements of the m. quadriceps of the dominant extremity was recorded in kilograms [14].

#### 2.2.5. Functional Capacity

The 6MWT was conducted following the criteria set by the ATS to assess functional capacity [15]. The subjects were instructed to walk as fast as possible between two cones in a 30 m corridor and the walking distance in 6 min was recorded in meters.

#### 2.2.6. Cognitive Function

The MoCA test was administered by a physiotherapist who had undergone training in both the application and scoring rules of the test to assess cognitive function. The maximum score attainable on the test is 30. A score of 21 and below was accepted as indicating mild cognitive impairment [16]. The Turkish reliability and validity study of the test was performed by Kaya et al. [17].

### 2.3. Statistical Analysis and Sample Size

Data were analyzed using the statistical software package IBM SPSS v.26 (SPSS Inc.; Chicago, IL, USA). The normality of the distribution of data were analyzed using the Kolmogorov−Smirnov test and the Shapiro–Wilk test. The comparison of categorical variables between groups was performed using the Chi-square test. The Independent *t*-test or Mann–Whitney U test was used for intergroup comparisons depending on the distribution properties of the data. The results were assessed within 95% confidence intervals (CIs), and Cohens’ d effect sizes were calculated. Pearson correlation analysis was used for normally distributed continuous data, and Spearman’ correlation analysis was used for ordinal or non-normally distributed data to assess the relationship of balance parameters with pulmonary function, respiratory and peripheral muscle strength, functional capacity, and cognitive function. A multiple linear regression model was used to assess which variables contributed to the prediction of the balance parameters. Variables presenting the highest correlation coefficient (r) with the balance parameters were included in the model. Variable selection was performed using the forward stepwise method. Only the model with the highest coefficient of determination (R^2^) is presented. A *p* value of < 0.05 was considered statistically significant for all analyses.

The sample size was calculated using the G*Power 3.1 (Universitaet Dusseldorf; Dusseldorf, Germany) [18]. Eymir et al. reported that there was a significant difference in the PST/Overall score assessed with the Biodex Balance System between the patients with COPD and healthy subjects [19]. The sample size calculation was made at 95% power and a two-tailed α level of 0.05 with the 1.404 effect size based on comparison of the PST/Overall score in patients with COPD and healthy subjects (0.96 ± 0.59 vs. 0.35 ± 0.17, respectively). Based on the provided calculation, we estimated a sample size of a minimum of 15 participants for each group. Participants were included in the study by calculating at least a 20% increase in sample size adjusting for the drop-out rate. 

## 3. Results

**Figure 2:**
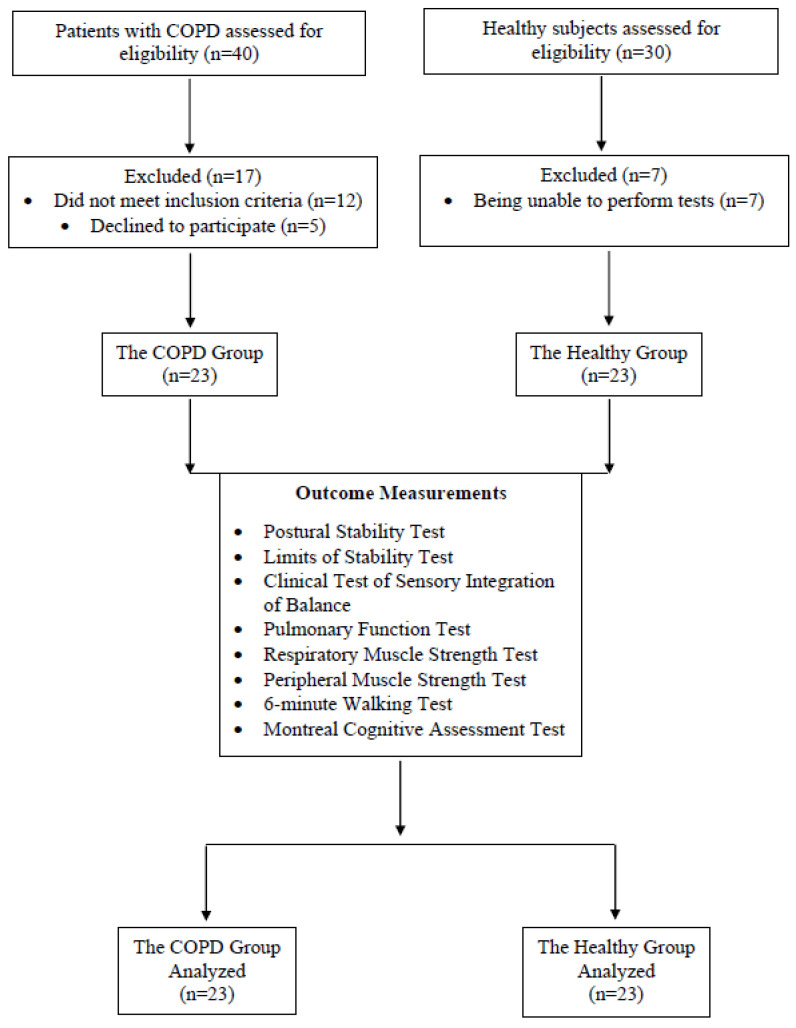
Flowchart of the study.

Forty patients with COPD were assessed for eligibility; a total of seventeen patients were excluded for not meeting the inclusion criteria or refusing to participate. Thirty age-matched healthy subjects were also assessed for eligibility; a total of seven subjects were excluded for being unable to perform tests. Twenty-three subjects for each group were included in the study and a total of forty-six participants completed the study with no drop-outs (Figure 2). The demographic and clinical characteristics of the participants are given in Table 1. No significant difference was found between the two groups’ demographic and clinical characteristics (*p* > 0.05).

There were significant differences in all outcome measures assessing balance, pulmonary function, respiratory muscle strength, peripheral muscle strength, and functional capacity in the COPD group compared to the healthy group (*p* < 0.05). There was no significant difference between the two groups in terms of the MoCA score (*p* > 0.05) (Table 2). Changes in cardiorespiratory responses during the 6MWT were similar except for changes in SpO_2_.

The PST/Overall score had a significant and strong correlation with MIP (r = −0.630, *p* = 0.001) and a significant and moderate correlation with m. quadriceps strength and 6MWT distance (r = −0.472, *p* = 0.023; r = −0.496, *p* = 0.016, respectively). LOST/Overall score and CTSIB/Composite score were not significantly correlated with any independent variable (*p* > 0.05) (Table 3).

The results of the multiple linear regression model for predicting the PST/Overall score are presented in Table 4. The variables of MIP, m. quadriceps strength, and 6MWT distance were included in the analysis. The results showed that MIP, m. quadriceps strength, and 6MWT distance were significant to explain the PST/Overall score with an R^2^ = 0.336 (*p* = 0.004). The standardized beta coefficients for MIP, m. quadriceps strength, and 6MWT distance were −0.319, −0.109, and −0.279, respectively.

## 4. Discussion

The findings of the present study demonstrated that the static and dynamic balance, pulmonary function, respiratory and peripheral muscle strength, and functional capacity of patients with COPD were impaired compared to age- and comorbidity-matched healthy subjects. One unanticipated finding was that there was no significant difference between cognitive function in the groups, but the MoCA score of both groups was below 21 points, indicating mild cognitive impairment [16]. It was also shown that functional capacity and inspiratory and peripheral muscle strength were the main determining factors for static balance. In contrast, dynamic balance outcomes did not have a significant relationship with any variable.

It is known that cerebral impairment and/or sensory reception and integration dysfunction caused by hypoxia-induced neuronal damage may play a role in impaired balance in patients with severe COPD [20]. In the limited number of previous studies investigating the relationship between pulmonary function and balance, no relationship between FEV_1_ and balance was found [21,22]. The authors attributed the lack of a significant correlation between pulmonary function and balance to the fact that the patients had relatively mild to moderate obstruction and there was not enough hypoxemia to impair balance. Our findings also showed that none of the pulmonary function variables was correlated with balance outcomes. The fact that the majority of the patients included in the present study were GOLD Stage II and III and none of them was receiving oxygen support may indicate that there was no presence of severe hypoxemia, and accordingly, pulmonary function may not have caused any problems that could affect the balance at the neuronal or motor level. To the best of our knowledge, there is only one study in which pulmonary function and balance were found to be related, and de Castro et al. reported that total lung capacity and static balance were correlated in this study and that impaired thoracic mechanics due to hyperinflation may have affected static balance [23]. Considering that 6MWT and mMRC score are correlated with hyperinflation [24], the relatively low mMRC score and high 6MWT distances of our patients can be interpreted as the reason for the low presence of hyperinflation and therefore the lack of correlation between balance and pulmonary function, even if we were not able to measure the total lung capacity, which primarily determines hyperinflation, in the present study.

In a recent study, predictors of postural control in patients with COPD were identified and MIP was found to be a significant predictor [25]. Impaired postural stability in patients with COPD has been shown to be associated with inspiratory muscle weakness and increased trunk muscle activity may limit the ability of the trunk to maintain static balance and increase the risk of falls [26]. Indeed, Yamada et al. reported that diaphragm movement during tidal breathing while standing was significantly higher and faster in patients with COPD compared to healthy subjects [27]. Our results showed that MIP was lower than in healthy subjects and it was only a significant determinant of static balance, whereas MEP was not correlated with either static or dynamic balance. Similarly, in the study in which static balance was evaluated with one-leg standing, a strong correlation was found with MIP, but no correlation was found with MEP [28]. Florian et al. found that improved MIP played a role in the enhancement in balance in patients with severe COPD in whom they provided inspiratory respiratory muscle training and thus revealed the relationship between MIP and balance [29]. Diaphragm contraction increasing intra-abdominal pressure and providing stability to the lower body during static postures is the most widely accepted theory that most clearly explains the direct relationship of MIP with static balance [30]. In our study, although the MEP value was lower compared to healthy controls, we found that the expiratory muscles could not be the determinant in either static or dynamic balance, which may be related to the fact that the expiratory muscles consist of muscle groups that are resistant to fatigue and have high endurance. This hypothesis is supported by Mesquita Montes et al., who showed that there was no difference between the abdominal muscle activities of patients with COPD and healthy controls during static and dynamic activities [31]. In addition, the potency of expiratory muscle metaboreflex has been reported to be lower and have a higher threshold compared to inspiratory muscle metaboreflex [32]. Considering the performance of the inspiratory and expiratory muscles required to maintain balance, the fact that expiratory muscles have physiologic properties that allow them to adapt easily may be another possible reason why our findings could not show any correlation between MEP and balance. 

In a recent study in which postural control was assessed with PST and LOST, it was found that patients with COPD had worse results compared to healthy subjects and that impaired postural control was directly related to muscle strength [33]. Consistent with previous findings, the current results showed that peripheral muscle strength and functional capacity were related to static balance [19,23,34]. The frequent use of oxygen therapy and hospitalizations, high level of dyspnea, and female gender were identified as possible mechanisms impairing balance in the studies in which dynamic balance was found to be related to exercise capacity and peripheral muscle strength [20,35]. Glucocorticoids, which are more frequently used during acute exacerbation and also in the chronic period, may decrease protein synthesis through the down-regulation of IGF-1, increase leptin, and cause activation of skeletal muscle mitochondrial-mediated apoptotic signaling pathways [36]. Since an exacerbation in the last 8 weeks was an exclusion criterion in our study, steroid-based medications may not have caused a loss of muscle mass and strength in patients. In the present study, only static balance was found to be related to exercise capacity and peripheral muscle strength, whereas dynamic balance was not. This may be related to the fact that none of the patients used oxygen support; most of them had GOLD stage II and III (73.9%) mild- to moderate-severity disease, had a relatively low mMRC score, and a low percentage of them were female (21.7%). Current studies reported that respiratory and hip muscles showed higher activation during static conditions in patients with COPD [23,26]. An optimal upright posture requires proprioceptive control, and the diaphragm, an important inspiratory muscle, plays a major role in stabilizing the spine during static posture. Increased respiratory workload and inspiratory function during exercise may alter the proprioceptive signals sent from the diaphragm and result in incomplete trunk stabilization [37]. The finding that MIP and 6MWT distance were independent determinants of static balance in our study showed that increased respiratory workload in patients with COPD may play a role in impaired balance by causing a decrease in both primary respiratory muscle strength and functional capacity in accordance with the literature.

Mild cognitive impairment is described as a clinical condition where there is a noticeable decrease in cognitive function beyond what is typical for a person’s age and level of education, yet it is not sufficiently severe to disrupt their activities of daily living [38]. Studies indicate that the level of cognitive impairment is increased in patients with COPD, especially those who have severe hypoxemia and require prolonged oxygen therapy [39]. Our results showed that while there was no significant difference in MoCA scores between the groups, there was mild cognitive impairment in both groups. Crişan et al. found that only patients with acute exacerbation of COPD had lower cognitive function than healthy controls and patients with stable COPD and stated that the inflammatory response during acute exacerbation may impair cognition [9]. The patients included in this study were also stable, did not use oxygen support, and had a relatively high rate of mild to moderate disease. Likewise, Van Hove et al. stated that the cognitive abilities of patients with COPD were not lower than healthy controls; in addition, they could not find a relationship between balance and cognitive function [8]. A scoping review has shown that cognitive capacity is reduced in older adults with COPD and that this reduction may be related with balance and peripheral muscle strength [40]. Although there are studies showing a relationship between cognitive function and balance [10,20], there are also studies presenting no relationship [8,41]. Therefore, this issue is still controversial and the number of studies is limited. In view of all, no relation was found in stable, mild to moderate, and relatively young patients, we may hypothesize that only in the presence of acute exacerbation and severe systemic inflammation can cognitive function deterioration reach a point where it creates balance impairment [42]. Given that the patients included in the present study were stable, relatively young, and had a similar level of cognitive function to the healthy subjects, this may explain why MoCA is not a determinant of balance.

The present study has certain limitations: Although the sample size was sufficient to detect differences between patients and healthy subjects in terms of balance-related factors, it might not have had enough statistical power when investigating the relationship between balance and its related factors. Another limitation is the relatively young age of the participants (64 years), even though we know that balance distribution has been strongly correlated with older age [7]. Considering the direct relationship between falls and balance and morbimortality in patients with COPD, the other important limitation is that the risk of falls could not be assessed. In addition, the fact that the patients included in our study were in moderate to severe stages of COPD limits the generalizability of our results for patients in mild and very severe stages. Finally, this study is limited by the lack of additional factors possibly related to balance in patients with COPD that were assessed, such as body composition, level of proprioception, and physical activity.

## 5. Conclusions

It has been reported that balance in patients with COPD is largely assessed with practical clinical tests, but assessments with laboratory-based sensitive and specific tests are insufficient [3]. The Biodex Balance System is a valid, reliable, and objective method to assess static and dynamic balance in older subjects [11]. To the best of our knowledge, this study is the first study in which balance in patients with COPD was assessed with a specific test and compared with healthy patients, and its relationship with the variables that may have the most association was investigated. The results of the present study indicate that the determinants of static balance patients with COPD are the maximum inspiratory pressure, knee extensor strength, and 6 min walking test distance, while dynamic balance could not be related to any variable. Additionally, this study revealed that balance is impaired in adults with COPD, even if compared with age- and comorbidity-matched healthy subjects. Therefore, for the management of the disease and reduction in falls in patients with COPD, we recommend including postural stability and static balance assessment and training of related variables in pulmonary rehabilitation.

## Figures and Tables

**Table 1:** The demographic and clinical characteristics of the participants.

	The COPD Group (*n* = 23)	The Healthy Group (*n* = 23)	*p* Value
**Age (years)**	64.26 ± 6.64	64.13 ± 4.83	0.947
**Gender**			0.730
Female	5 (21.7%)	6 (26.1%)	
Male	18 (78.3%)	17 (73.9%)	
**Body composition**			
Weight (kg)	73.55 ± 13.40	73.91 ± 8.90	0.758
Height (cm)	169.43 ± 6.06	166.53 ± 8.79	0.349
Body mass index (kg/m^2^)	25.54 ± 4.02	26.71 ± 2.47	0.404
**Smoking history (pack/year)**	35.74 ± 13.62	28.70 ± 13.38	0.072
**Comorbidities**			
Hypertension	11 (47.8%)	10 (43.4%)	0.618
Diabetes	7 (30.4%)	5 (21.7%)	
Hyperlipidemia	5 (21.7%)	4 (17.3%)	
Sarcopenia	8 (34.7%)	6 (26.0%)	
**Disease duration (year)**	9.48 ± 7.80	N/A	N/A
**GOLD stage**		N/A	N/A
I	0		
II	9 (39.1%)		
III	8 (34.7%)		
IV	4 (26.1%)		
**mMRC**	1.74 ± 0.81	N/A	N/A
**Oxygen support/NIV device**	0	N/A	N/A

Data are presented as mean ± standard deviation or *n* (%). Abbreviations: BMI: nody mass index; cm: centimeter; GOLD: Global Initiative for Chronic Obstructive Lung Disease; kg: kilogram; kcal: kilocalories; m: meter; mMRC: Modified Medical Research Council Scale; NIV: noninvasive mechanical ventilation; N/A: not applicable.

**Table 2:** Comparison of the balance, pulmonary function, respiratory muscle strength, peripheral muscle strength, functional capacity, and cognitive function between the groups.

	The COPD Group (*n* = 23)	The Healthy Group (*n* = 23)	*p* Value	95% Cl		Effect Size (Cohen’s d)
				Lower	Upper	
**Balance**						
PST/Overall	0.52 ± 0.21	0.28 ± 0.07	<0.001 *	0.14	0.33	1.533
LOST/Overall	36.34 ± 12.32	57.39 ± 3.47	<0.001 *	−26.53	−15.55	2.325
CTSIB/Composite score	1.90 ± 0.12	1.09 ± 0.14	<0.001 *	0.73	0.89	6.212
**Pulmonary function**						
FVC (% pred)	65.82 ± 18.98	76.39 ± 5.18	0.016 *	−19.01	−2.11	0.759
FEV_1_ (% pred)	43.52 ± 15.76	72.39 ± 5.70	<0.001 *	−36.03	−21.70	2.436
FEV_1_/FVC (%)	51.51 ± 7.98	85.43 ± 3.96	<0.001 *	−38.00	−30.43	5.384
PEF (% pred)	39.82 ± 13.60	77.39 ± 5.70	<0.001 *	−43.85	−31.27	3.603
FEF_25–75_ (% pred)	21.86 ± 8.95	70.30 ± 6.16	<0.001 *	−53.01	−43.85	6.305
**Respiratory muscle strength**						
MIP (cmH_2_O)	71.52 ± 16.39	87.21 ± 4.90	0.022 *	−16.03	1.63	0.870
MEP (cmH_2_O)	84.47 ± 36.71	101.82 ± 12.48	0.041 *	−33.93	−0.75	0.632
**Peripheral muscle strength**						
M. quadriceps (kg)	37.29 ± 3.76	46.56 ± 6.68	<0.001 *	−12.51	−6.02	1.710
**Functional capacity**						
6MWT distance (m)	458.13 ± 104.58	586.69 ± 28.85	<0.001 *	−175.12	−82.00	1.675
ΔSpO_2_ (%)	−3.73 ± 3.17	−2.01 ± 1.19	0.030 *	−1.72	−1.05	0.718
ΔHR (bpm)	41.13 ± 15.94	37.47 ± 21.56	0.061	3.66	16.52	0.193
ΔDyspnea (MBS)	1.82 ± 1.15	1.70 ± 0.99	0.077	0.12	0.85	0.111
ΔFatigue (MBS)	1.30 ± 1.06	1.33 ± 1.01	0.890	0.03	0.67	0.028
**Cognitive function**						
MoCA score	18.39 ± 4.97	20.57 ± 0.99	0.052	−4.36	0.01	0.287

* Statistically significant (*p* ≤ 0.05). Data are presented as mean ± standard deviation. Abbreviations: 6MWT: 6 min walking test; bpm: beats per minute; cm: centimeter; CTSIB: clinical test of sensory integration of balance; FEF_25–75:_ forced mid-expiratory flow between 25% and 75%; FEV_1:_ forced expiratory volume in 1 s; FVC: forced vital capacity; HR: heart rate; kg: kilogram; LOST: limits of stability test; m: meter; MBS: modified Borg scale; MEP: maximal expiratory pressure; MIP: maximal inspiratory pressure; MoCA: Montreal Cognitive Assessment; PEF: peak expiratory flow; pred: predicted; PST: postural stability test; SpO_2_: oxygen saturation.

**Table 3:** Correlation of pulmonary function, respiratory muscle strength, peripheral muscle strength, functional capacity, and cognitive function with balance.

	PST/Overall		LOST/Overall		CTSIB/Composite Score	
	r	*p*	r	*p*	r	*p*
**Pulmonary function**						
FVC (% pred)	−0.412	0.051	0.320	0.137	−0.241	0.267
FEV_1_ (% pred)	−0.371	0.082	0.332	0.122	−0.246	0.258
FEV_1_/FVC (%)	0.086	0.698	0.051	0.817	0.022	0.920
PEF (% pred)	0.288	0.182	0.190	0.368	0.206	0.346
FEF_25–75_ (% pred)	−0.051	0.817	0.166	0.449	−0.204	0.350
**Respiratory muscle strength**						
MIP (cmH_2_O)	−0.630	0.001 *	−0.071	0.747	0.084	0.702
MEP (cmH_2_O)	−0.324	0.132	0.153	0.485	−0.042	0.849
**Peripheral muscle strength**						
M. quadriceps (kg)	−0.472	0.023 *	0.123	0.575	0.160	0.464
**Functional capacity**						
6MWT distance (m)	−0.496	0.016 *	0.237	0.276	−0.058	0.794
**Cognitive function**						
MoCA score	−0.335	0.118	0.003	0.990	0.005	0.984

* Statistically significant (*p* ≤ 0.05). Abbreviations: 6MWT: 6 min walking test; cm: centimeter; CTSIB: clinical test of sensory integration of balance; FEF_25–75:_ forced mid-expiratory flow between 25% and 75%; FEV_1:_ forced expiratory volume in 1 s; FVC: forced vital capacity; kg: kilogram; LOST: limits of stability test; m: meter; MEP: maximal expiratory pressure; MIP: maximal inspiratory pressure; MoCA: Montreal Cognitive Assessment; PEF: peak expiratory flow; pred: predicted; PST: postural stability test.

**Table 4:** Multiple linear regression model for predicting the postural stability test/overall score.

	B	Standard Error of B (95% CI)	Standardized Beta Coefficient	*p*	R^2^
Constant	1.324	0.405 (0.477–2.171)		0.004 *	0.336
MIP (cmH_2_O)	−0.004	0.003 (−0.010–0.002)	−0.319		
M. quadriceps (kg)	−0.006	0.012 (−0.032–0.020)	−0.109		
6MWT distance (m)	−0.001	0.000 (−0.002–0.000)	−0.279		

* Statistically significant (*p* ≤ 0.05). Abbreviations: 6MWT: 6 min walking test; cm: centimeter; kg: kilogram; MIP: maximal inspiratory pressure; PST: postural stability test.

## Data Availability

Data is unavailable due to privacy and ethical restrictions.
